# Life as an early career researcher: interview with Yu Shrike Zhang

**DOI:** 10.4155/fsoa-2017-0114

**Published:** 2017-11-15

**Authors:** Yu Shrike Zhang

**Affiliations:** 1 Division of Engineering in Medicine, Department of Medicine, Brigham & Women’s Hospital, Harvard Medical School, Cambridge, MA 02155, USA

## Abstract

**Yu Shrike Zhang talks to Francesca Lake, Head of Open Access Publishing:** Yu Shrike Zhang received his BE degree in biomedical engineering in 2008 from Southeast University, PR China and PhD degree in biomedical engineering in 2013 from Georgia Institute of Technology. He is currently an Instructor of Medicine and Associate Bioengineer in the Division of Engineering in Medicine at the Brigham and Women’s Hospital, Harvard Medical School. His research interests include organs-on-chips, biomaterials, bioprinting, biomedical devices, biomedical imaging and biosensing with a focus on innovating medical engineering technologies to recreate functional tissues and biomimetic tissue models. He was also a finalist in the inaugural Future Science Early Career Research Award.

## Can you tell us a little about your career path to date?

Sure. I received my BE degree from the School of Biological Science & Medical Engineering at Southeast University, China in 2008. Then I came to the USA to pursue doctoral studies, where I further obtained my MS from the Department of Biomedical Engineering at Washington University in St Louis in 2011 and PhD from the Wallace H Coulter Department of Biomedical Engineering at Georgia Institute of Technology and Emory University School of Medicine in 2013. After a postdoctoral training period at the Brigham and Women’s Hospital at Harvard Medical School, I am currently an Instructor of Medicine at Harvard Medical School and Associate Bioengineer at the Brigham and Women’s Hospital.

## What are you working on at the moment?

My research is centered on innovating medical engineering technologies to recreate functional biomimetic tissues and tissue models, including 3D bioprinting, organs-on-chips, biomedical devices, biomedical imaging, biosensing and cancer theranostics. We are actively collaborating with a multidisciplinary team encompassing biomedical, mechanical, electrical and computer engineers as well as biologists and clinicians to ultimately translate these cutting-edge technologies into clinics.

## What do you find most rewarding about your work?

The most rewarding thing about my work is when the challenges we face in our research are overcome through endeavor and when we see the potential of our technologies in changing the way that people in the field practice.

For example, the organ-on-a-chip platforms that we are building will likely bridge the gap between the conventional models (planar static cultures or animals) and the human system, by providing improved biomimetic *in vitro* microenvironments using a combination of 3D engineered tissue models and dynamic stimulation. Our own endeavors, along with those from other research groups on these organ-on-a-chip platforms, have the potential to transform the landscape of drug screening and personalized medicine.

## And what do you find the most challenging?

What I have found the most challenging throughout my academic career has been to define scientific or engineering problems – this requires one to understand the field very well and know where the niches are. Furthermore, once the problems are defined, the challenge becomes how to solve them using the most rigorous yet simplest ways.

## In your application to the Future Science Early Career Research Award, you discussed how you had to change your research direction with little guidance, which you have turned into a great success. Why & how did you do this, & what did this experience teach you?

Well, this is the fun part of research. Nothing is static and science is always evolving. Before, I was more into the use of biomaterials to engineer functional tissue substitutes for applications in regenerative medicine, but later on shifted my direction to the use of these technologies to generate biomimetic tissue models for personalized drug screening purposes, which I felt was closer to clinical translation than the former. Of course, there were many challenges associated during this transition but the way of doing research is similar – dedication, critical thinking and constant learning. These experiences in different areas are precious as they have broadened my vision and are certainly beneficial especially for an early-stage investigator like myself.

## What particular challenges did you face during this transition?

The main challenge has been the integration of microfluidics, which was an area that I was not very familiar with before I transitioned into this new field, to the engineered tissue models so that we could introduce dynamic microenvironments that better mimic the human physiology. Not only that, we have also had a hard time in developing a series of microfluidic biosensors to include in our organ-on-a-chip systems to achieve *in situ*, real-time and continual measurements of biophysical/biochemical parameters in a noninvasive manner, which took quite some effort.

## You have mentored a large number of students. What are the main pieces of advice you give them?

That love in what they do is always the most important. That is followed by perseverance – experiments fail all the time, and successes are rare in research. I have seen many examples where students just give up in the beginning and are unwilling to report failed data. But, practice and critical thinking make for perfection too – the more they practice, and the more they really think about their failures, the more successful they will become.

## What would you say makes a good mentor?

Patience is the key, as well as the courage to face failures together with your mentees – again, in the scientific world, failures are much more frequent than successes, and this is something a mentor should clearly understand too. A good mentor in my view should also balance the encouragement and stresses that he/she exerts on the mentees to best guide them into the formation of the mentality that self-motivation is always what drives successes in science, not external forces.

## If you could go back in time to when you launched your career, what advice would you give to yourself?

This is a tough question. If I had to choose one, maybe I would have urged myself to focus harder on learning fundamental knowledge – they all turned out to be quite useful throughout the course of my career.

## What are the main challenges facing early career researchers, & how would you go about tackling them?

I would say how to balance interest and reality is probably challenging. There are many intriguing ideas and areas that I want to explore, but at an early career stage, funding and energy are both limited and you have to choose the most important niches to start with.

## Finally, you are currently an Instructor of Medicine. What would you say your next steps are?

Further advancement into the next stage I guess – hopefully would not be too far away. This would certainly require continued research excellence and more importantly at this stage, the ability to gather more research funding that could support the activities of the laboratory into its further growth.

